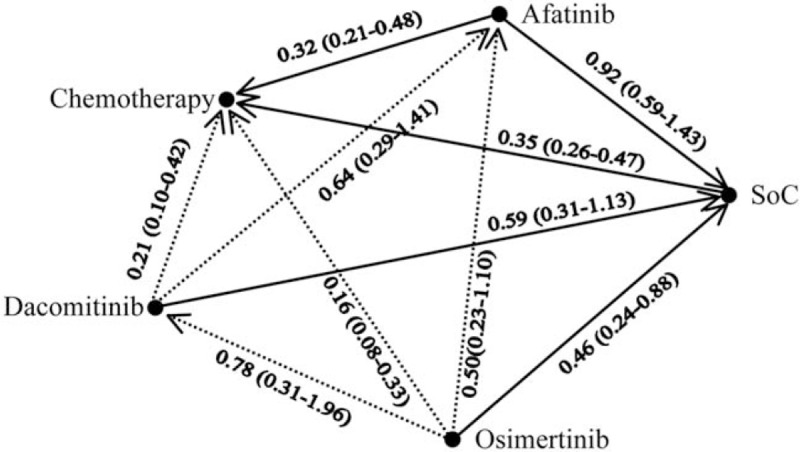# A network meta-analysis of nonsmall-cell lung cancer patients with an activating EGFR mutation: Should osimertinib be the first-line treatment?: Erratum

**DOI:** 10.1097/MD.0000000000016824

**Published:** 2019-08-09

In the article, “A network meta-analysis of nonsmall-cell lung cancer patients with an activating EGFR mutation: Should osimertinib be the first-line treatment?”,^[1]^ which appeared in Volume 97, Issue 30 of *Medicine*, several corrections need to be noted based on corrections to the hazard ratios (HR) and confidence intervals (CI) for the subgroup of men, non-Asians, smokers and Del19 mutations with new ln-derived values.

In the Results section of the Abstract, the sentence starting with “Compared with erlotinib or gefitinib, osimertinib was…” should needs to be corrected to “Compared with erlotinib or gefitinib, osimertinib was associated with improvement in men (HR = 0.46, 95% CI, 0.24–0.88), non-Asians (HR = 0.34, 95% CI, 0.12–0.95), smokers (HR = 0.48, 95% CI, 0.26–0.88), and those with a Del19 mutation (HR = 0.43, 95% CI, 0.24–0.78).”

In the Results section of the paper, the sentence starting with “Regarding PFS, compared with SoC, the 3 TKIs with…” should be corrected to “Regarding PFS, compared with SoC, the three TKIs with the highest probability of benefit were osimertinib, dacomitinib, and afatinib, with HRs (95% CI) of 0.46 (0.24–0.88), 0.59 (0.31–1.13), and 0.92 (0.59–1.43), respectively.” Also, the sentence starting with “Compared with SoC, osimertinib was associated with improvement in men…” should be corrected to “Compared with SoC, osimertinib was associated with improvement in men (HR = 0.46, 95% CI, 0.24–0.88), non-Asians (HR = 0.34, 95% CI, 0.12–0.95), smokers (HR = 0.48, 95% CI, 0.26–0.88), and those with a Del19 mutation (HR = 0.43, 95% CI, 0.24–0.78).”

In Table 3, all values which were log10-derived in the published version were changed to ln-derived in the corrected version.

**Table 3:**
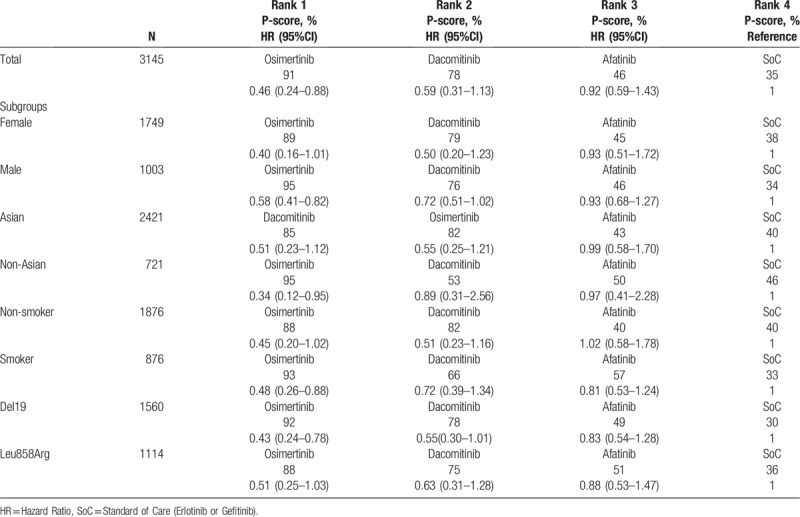
Rank and P-scores of subgroups.

In Figure 2, the line between dacomitinib and SoC should be a solid line and was a dash line before correction.

**Figure:**